# Evolutionary consequences of vector-borne transmission: how using vectors shapes host, vector and pathogen evolution

**DOI:** 10.1017/S0031182022001378

**Published:** 2022-11

**Authors:** Daniela de Angeli Dutra, Robert Poulin, Francisco C. Ferreira

**Affiliations:** 1 Department of Zoology, University of Otago, PO Box 56, Dunedin, New Zealand; 2 Center for Vector Biology, Rutgers University, New Brunswick, USA

**Keywords:** Coevolution, evolution, parasite–host interaction, vector-borne diseases, vector ecology, virulence

## Abstract

Transmission mode is a key factor that influences host–parasite coevolution. Vector-borne pathogens are among the most important disease agents for humans and wildlife due to their broad distribution, high diversity, prevalence and lethality. They comprise some of the most important and widespread human pathogens, such as yellow fever, leishmania and malaria. Vector-borne parasites (in this review, those transmitted by blood-feeding Diptera) follow unique transmission routes towards their vertebrate hosts. Consequently, each part of this tri-partite (i.e. parasite, vector and host) interaction can influence co- and counter-evolutionary pressures among antagonists. This mode of transmission may favour the evolution of greater virulence to the vertebrate host; however, pathogen–vector interactions can also have a broad spectrum of fitness costs to the insect vector. To complete their life cycle, vector-borne pathogens must overcome immune responses from 2 unrelated organisms, since they can activate responses in both vertebrate and invertebrate hosts, possibly creating a trade-off between investments against both types of immunity. Here, we assess how dipteran vector-borne transmission shapes the evolution of hosts, vectors and the pathogens themselves. Hosts, vectors and pathogens co-evolve together in a constant antagonistic arms race with each participant's primary goal being to maximize its performance and fitness.

## Graphical abstract

**Figure:**
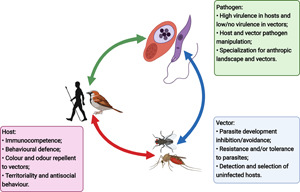

## Introduction

Symbionts live with or within their hosts and represent one of the most successful life-history strategies (Mestre *et al*., 2020). Due to their evolutionary success, virulent symbionts (i.e. pathogenic parasites) such as protozoans, helminths, bacteria and viruses probably account for over half of the world's biodiversity (Clayton *et al*., 2015). Indeed, a parasitic mode of life has evolved independently multiple times into variable life-history strategies that include fecal–oral, trophic transmission, airborne transmission and the use of vectors (i.e. mobile blood-feeding invertebrates involved in the transmission of pathogens to new potential hosts) (Weinstein and Kuris, 2016; Wilson *et al*., 2017). Vector-borne pathogens cause many of the most important infectious diseases that plague humans and animal hosts (Table 1) and will continue to do so in the next decades due to effects of climate change on arthropod vectors' abundance and distribution (Kelly-Hope *et al*., 2009; Garamszegi, 2011; Pérez-Rodríguez *et al*., 2014). Vector-borne pathogens include phylogenetically unrelated symbionts whose reliance on vectors emerged independently; hence, vector-borne pathogens present several distinct developmental strategies within their vectors that are reflected in many independent evolutionary histories among hosts, vectors and pathogens.

**Table 1.** Pathogen name, pathogen and vector type and number of deaths and cases for the main human vector-borne diseases

Disease	Pathogen name	Pathogen type	Vector type	Deaths per year	Cases per year	Geographical distribution
Malaria	*Plasmodium* spp.	Protozoa	Mosquito (*Anopheles*)	~600 000	200–400 million	Africa, tropical and subtropical parts of Asia and Oceania, Latin America
Dengue	Dengue virus	Virus	Mosquito (*Aedes*)	~40 000	50–100 million	Africa, tropical and subtropical parts of Asia and Oceania, Latin America
Leishmaniasis	*Leishmania* spp.	Protozoa	Sandfly	~30 000	~1.3 million	Africa, tropical and subtropical parts of Asia, Americas
Yellow fever	Yellow fever virus	Virus	Mosquito (*Aedes*)	~30 000	~120 000	Africa and Latin America
Japanese encephalitis	Japanese encephalitis virus	Virus	Mosquito (*Culex*)	10 000–20 000	~50 000	East Asia
Chagas disease	*Trypanosoma cruzi*	Protozoa	Kissing bug	~10 000	~7–8 million currently infected	Latin America
Lymphatic filariasis	Microfilaria	Nematode	Mosquito	<1000	~36 million currently infected	Africa, Asia, Americas and Pacific Islands
Chikungunya	Chikungunya virus	Virus	Mosquito (*Aedes*)	<1000	200 000–600 000	Mostly Asia and Americas
Lyme disease	Borrelia bacteria	Bacteria	Tick	<1000	300 000–400 000	North America, Europe and North Asia
Crimean-Congo haemorrhagic fever	Crimean-Congo haemorrhagic fever virus	Virus	Tick	30% infected people	Variable	Africa, West Asia and East Europe

*Source*: World Health Organization (WHO, 2014, 2020) and Centre of Disease Control and Prevention (CDC).

Pathogens are subjected to several evolutionary selective pressures that are intrinsically dependent on their mode of transmission and dispersal ability (Ewald, 1995; Frank, 1996; Powell, 2019). For example, pathogens transmitted by mobile vectors might evolve towards phenotypes of higher virulence (i.e. extent of damage that a consumer inflicts to organisms being exploited) against the vertebrate host than related pathogens that rely on dispersal *via* a single host (Ewald, 1983; Day, 2002). Other differences in pathogen life cycle may also modulate host–parasite evolution (Frank, 1996; Powell, 2019; Mestre *et al*., 2020). For instance, many trophically transmitted parasites benefit from an infection-induced increase in their host vulnerability to predation (Moore, 2002; Poulin *et al*., 2005) as it enhances their chance of transmission to their next host. For this reason, evolution favoured trophically transmitted parasites that could manipulate their hosts' behaviour to specifically increase their risk of predation (Moore, 2002). Horizontally transmitted pathogens (i.e. pathogens transmitted among hosts outside the strict parent–offspring relationship) are subjected to a trade-off between increasing their reproduction and keeping their host alive, since increases in pathogen replication are generally associated with greater virulence through more aggressive exploitation of resources that can kill the host (Ewald, 1983; Giorgio, 1995; Frank, 1996; Davies *et al*., 2001). Therefore, pathogen selection should favour a balance between replication and virulence that leads to the highest lifetime transmission success.

Among horizontally transmitted pathogens, those transmitted by vectors face unique trade-offs because they must invade, escape immune defences and be transmitted between 2 phylogenetically distant organisms (i.e. hosts and vectors), with distinct immune systems. Here, we explore the evolutionary pressures and consequences of the use of dipteran vectors for the pathogens, hosts and the vectors themselves, hereby unifying these 3 components in a common framework. By applying this framework, we aim to identify potential gains and deleterious effects of co- and counter-evolution among the host–vector–pathogen triad to highlight trends in host–vector–pathogen evolution. We focus mostly on vertebrate hosts, such as mammals and birds, which sustain pathogen development and transmission *via* haematophagous dipteran vectors. Then, we discuss how each component of this triad influences the selective evolutionary pressures acting on the 2 other counterparts and propose new research directions.

## Evolutionary consequences of vector transmission for vertebrate hosts

Certain host species’ traits and individual behaviours can be associated with infection risk by vector-borne pathogens, such as body size and preening behaviour (Bush and Clayton, 2018; Filion *et al*., 2020). For mosquitoes (family Culicidae) and sand flies (family Psychodidae), olfactory cues seem to be the main driver of host detection. Several studies have investigated the effects of odours and identified many odourants positively or negatively related to vector attraction (Lucas-Barbosa *et al*., 2021; Yan *et al*., 2021). In addition, carbon dioxide (CO_2_) has been recognized as one of the most important olfactory cues for host-seeking behaviour (Pinto *et al*., 2001; Müller *et al*., 2015; Yan *et al*., 2021). Larger body sizes emit greater volumes of carbon dioxide, increasing vector attractiveness (Daviews *et al*., 1991; Yan *et al*., 2018). Similar relationships exist for body temperature, as higher body temperatures also lead to higher emission of carbon dioxide (van Loon *et al*., 2015).

While vectors rely mainly on olfactory cues to locate hosts at long distances, visual cues are markedly important for short-range host choice (Cardé and Gibson, 2010). For example, coloration patterns can influence vector landing on their hosts, and there is evidence showing that darker colours are more attractive to mosquitos compared to lighter ones (Yan *et al*., 2021). Nonetheless, contrast against the background seems to be a more important cue for vector attraction than colours and intensity on their own (Yan *et al*., 2021). On the other hand, stripes seem to confer protection for the host. How *et al*. (2020) demonstrated that tabanid flies attempting to approach horses dressed in striped rugs remained more distant from the target and achieved lower landing success than flies approaching horses wearing black or grey rugs. However, the mechanism by which stripes protect zebras from these vectors is still poorly understood. Since vector attraction is shaped by host features that vary within species such as odour, size (Lucas-Barbosa *et al*., 2021) and colour (Yan *et al*., 2021), host individuals presenting traits less attractive or even repellent to vectors could benefit from lower pathogen exposure, potentially achieving higher fitness compared to their infected counterparts. Those traits (e.g. coloration and body size) are also subject to other selection pressures such as mating success, intraspecific competition and predator avoidance; therefore, selection for anti-vector traits should be balanced against selection for other fitness-enhancing functions.

Hosts from the same species may possess variable attractiveness to vectors. Prasadini (2019) suggested that *Aedes aegypti* mosquitoes fed preferably on people belonging to the blood type ‘O’ and that the blood type ‘A’ may confer protection against some diseases, such as dengue and zika, as people classified in this group incur the lowest biting rates. On the other hand, Goel *et al*. (2015) have shown that *Plasmodium falciparum* binds preferably to blood group ‘A’ cells, increasing formation of rosettes, severity of infection and potentially contributing to the heterogeneous distribution of ABO blood groups worldwide by favouring blood group ‘O’. Malaria is among the strongest evolutionary pressures in late human history (Hedrick, 2011) and, as a result, vectors and pathogens exert pressure on their vertebrate hosts through distinct pathways that drive the selection of distinct host phenotypes.

Anti-pathogen and anti-vector behaviours such as preening/grooming, scratching and nest maintenance are commonly observed in nature (Bush and Clayton, 2018; Sarabian *et al*., 2018; Poulin *et al*., 2020). Pathogen avoidance strategies can be costly to their hosts since they demand resources and may cause hosts to miss out on foraging and mating opportunities (Poulin *et al*., 2020). Preening/grooming behaviour is an effective strategy used by animals to control ectoparasite load and possibly avoid vector-borne infections. However, this does not necessarily reduce infection risk by pathogens transmitted by those vectors (Waite *et al*., 2014). At the same time, for animals socially organized into groups or colonies, preening/grooming of potential vectors (e.g. flies) might increase general pathogen prevalence. This may happen because host cleaning can induce vectors to move to new host individuals, increasing pathogen dissemination within the colony (Bush and Clayton, 2018). Moreover, the undue annoyance and vigorous swatting behaviour displayed by many animals are disproportionate to the amount of blood removed by the insect and the effect of the blood loss on fitness. In other words, the direct fitness loss associated with blood feeding by the occasional vector is often smaller than the indirect fitness loss associated with pathogen transmission; hence the latter is expected to exert a much stronger selective pressure for the host.

Hosts should evolve towards phenotypes of pathogen resistance (i.e. host capability to limit pathogen proliferation) or tolerance (i.e. host capability to reduce pathogenic effects of infection without controlling pathogen load/burden) depending on the cost of infection (Singh and Best, 2021). Indeed, introduction of the avian malaria parasite *Plasmodium relictum* has seemingly driven evolution of the Hawaiian honeycreeper amakihi *Chlorodrepanis virens* by selecting resistant/tolerant populations due to the strong selective pressure exerted by the parasite (Atkinson *et al*., 2013). In this case, immune-related genes were inferred to be under selection in areas with high rates of *Plasmodium* transmission (Cassin-Sackett *et al*., 2019). At the same time, avian malaria has been a major cause of extinction and population declines in the Hawaiian Islands (Van Riper *et al*., 1986; Lapointe *et al*., 2012), indicating that evolution of tolerance to this novel pathogen does not occur for all bird species. Since vector-borne pathogens are generally more virulent than other pathogens (Ewald, 1983, 1995; Frank, 1996), they may exert stronger selective pressures driving host evolution (Woolhouse *et al*., 2002). A classic example of host counter-evolution to vector-borne parasites is the high prevalence of the sickle cell haemoglobin gene in highly endemic human malaria regions in Africa (Hedrick, 2011). This gene induces malformation of red blood cells and, consequently, weakens the ability of cells to transport oxygen. In these regions, however, the benefit arising from malaria resistance surpasses the deleterious effects due to lower oxygen transport, which allows the maintenance of high frequencies of the sickle cell haemoglobin gene in human populations (Hedrick, 2011). Vector-borne pathogens should promote the evolution of protective host phenotypes (e.g. low vector attraction, high tolerance to infection) which are shaped by pathogens, vectors and other biotic and abiotic (i.e. interaction with other organisms and environmental conditions, respectively) selective pressures over evolutionary time.

## Evolutionary consequences of vector transmission for vectors

As presented above, theoretical and empirical data support the notion that vector-borne pathogens can pose high costs to their vertebrate host. However, what are the pathogen replication/virulence trade-offs in relation to transmission success from the vector's perspective? *Plasmodium* parasites may reduce either vector survivorship (Ferguson and Read, 2002; Lambrechts and Scott, 2009) or fertility (Pigeault and Villa, 2018). Nevertheless, it is often difficult to estimate whether the presence of blood parasites decreases vector fitness and survivorship by direct deleterious effects or as a mere consequence of lower quality of the infected blood (Ferguson *et al*., 2003*b*; Kotepui *et al*., 2014; Pigeault *et al*., 2015). However, infection by some avian *Plasmodium* can increase vector survivorship (Vézilier *et al*., 2012; Gutiérrez-López *et al*., 2020), a phenotypic alteration that favours parasite transmission. Although mosquito-borne viruses can be pathogenic to their vectors (Girard *et al*., 2005) and may change their behaviour (Jackson *et al*., 2012), these effects are generally subtle (Halbach *et al*., 2017). Broadly, Alphaviruses with horizontal transmission (e.g. *via* blood feeding on infected hosts) are likely to increase mortality in vectors, whereas Bunyaviruses vertically transmitted within *Aedes* mosquitoes from females to their progeny do not induce mortality in the vector (Lambrechts and Scott, 2009). This happens due to the increased selective pressure that vertically transmitted pathogens face to not harm their vectors (Ebert, 2013).

Pathogens may directly harm their vectors by tissue damage, through activation of the immune system to fight off the infection or by subtracting resources for their own development and replication (Shaw *et al*., 2022). However, these effects were shown to be subtle at the transcriptome level in interactions between *Culex* and avian malaria parasites likely to occur in Hawaiʻi (Ferreira *et al*., 2022). *Leishmania* parasites cause structural damages in the sand fly (*Lutzomyia longipalpis*) gut (Schlein *et al*., 1992), reducing vector longevity without affecting its fecundity (Rogers and Bates, 2007). Therefore, vectors, similarly to vertebrate hosts, would benefit from the evolution of mechanisms that limit either pathogen multiplication (i.e. resistance) or the costs associated with response to the infection (i.e. tolerance). Alternatively, uninfected vectors could avoid feeding on infected hosts if the pathogen is costly to the vectors themselves. This parasite avoidance behaviour has been demonstrated in fewer studies (see Lalubin *et al*., 2012) when compared to a larger body of studies showing higher vector attraction to infected hosts (Cozzarolo *et al*., 2020; Santiago-Alarcon and Ferreira, 2020). Nonetheless, some earlier studies also suggest the absence of any effect of infection status on vector attraction (Cozzarolo *et al*., 2022).

There seems to be a threshold for parasite density within the host at which stochasticity determines the chances of a vector becoming infected (Alizon and van Baalen, 2008). In human malaria, few mosquitoes become infected with *Plasmodium vivax* and *P. falciparum* after taking an infectious blood meal, and infection rates are positively correlated with parasite density in the blood source (Nguitragool *et al*., 2017; Tadesse *et al*., 2018). Few highly susceptible mosquitoes of the same *Anopheles* species harbour high parasite burdens when infected with *Plasmodium* parasites, while most individuals carry only a few oocysts, creating the general overdispersed pattern with a low median number (1–4) of oocysts per mosquito (Bompard *et al*., 2020; Graumans *et al*., 2020). In the case of *Leishmania* parasites, hosts carrying the greatest parasitaemia levels are primarily responsible for vector (sand flies) infection, which in turn will be more likely to infect another vertebrate host (Miller *et al*., 2014). The overall variability in parasite infection rate and burden among vector specimens vary according to the amount of parasite ingested, which is a factor of blood meal size and parasitaemia (Da *et al*., 2015; Emami *et al*., 2017). However, little is known about how individual vector factors such as immune response affect parasite burden, individual mosquito susceptibility to parasite infection and the vector's ability to prevent pathogen development.

High parasite loads might result in vector death (Dawes *et al*., 2009). Consequently, vectors might evolve towards pathogen inhibition. For instance, mosquitoes can arrest the development of *Plasmodium* ookinetes and oocysts by melanotic encapsulation (i.e. deposition of melanin on the surface of invading pathogen) or by cell lyses as ookinetes cross the midgut (Beier, 1998; Hoffmann *et al*., 1999; Wen-Yue *et al*., 2007). Vectors can also constrain parasite development by degrading sporozoites when these migrate to the salivary glands through the haemolymph (Hillyer *et al*., 2007). At the same time, development of the pathogens in non-competent vectors can induce very high insect mortality rates. Valkiunas *et al*. (2014) showed that the avian malaria-like parasites *Haemoproteus* spp., whose vectors are *Culicoides* biting midges, kill mosquitoes that feed on birds with high parasite loads even in such abortive infections. However, low parasite burdens in the vertebrate host do not reduce mosquito survival. Therefore, vector avoidance towards hosts infected with deadly pathogens, or inhibition strategies against such pathogens within-vectors, should have been selected over the course of vector–host–pathogen evolution.

Although most research has focused on the impact of parasites on vector biology, vertebrate hosts also evolve behavioural responses and strategies to avoid or suppress vector blood meals (Billingsley *et al*., 2006). Therefore, vectors should evolve to minimize risks of being killed by the vertebrate host. Indeed, vectors have developed several mechanisms to avoid host defensive behaviours. Nocturnal vectors could benefit from feeding on diurnal hosts, while diurnal vectors would benefit from feeding on nocturnal hosts. For instance, Killeen *et al*. (2006) observed that about 80% of interactions between people and *Anopheles* mosquitoes occurred during peak sleeping hours. Feeding when hosts are not active is an advantageous behaviour for vectors because it allows the vectors to avoid behavioural defences. In addition, during blood ingestion, mosquitoes inject vasodilatory, antiplatelet and anti-inflammatory chemicals to reduce their detectability (Billingsley *et al*., 2006). Together with a blood meal, vectors ingest host immunoglobulins and proteins from the complement system which remain active from a couple of hours to days; these can have deleterious effects, causing reduction in fitness and survival or even death of vectors (Maitre *et al*., 2022). The host skin microbiome alters vector preference towards individual hosts and these microbes can also modulate host immune responses (Naik *et al*., 2012). Nevertheless, very little research has yet examined whether host defences are a driver of vector specialization/evolution. Additionally, future studies should investigate the potential association between vertebrate skin microbes and their role in host's immune response against vectors and vector fitness itself. Overall, vectors should benefit from and should evolve towards strategies to avoid pathogen infection, reduce infection damage, inhibit pathogen development and overcome host behavioural and immune defences.

## Evolutionary consequences of vector transmission for pathogens

Pathogens can benefit from the use of vectors since it can increase pathogen transmissibility and spatial dispersal due to vector mobility. These advantages occur when the supply of vectors is greater than the supply of vertebrate hosts (Ewald, 1995; Auld and Tinsley, 2015) since mosquitoes can act as both reservoirs and vectors, maintaining and spreading the infection (Santiago-Alarcon *et al*., 2012). Pathogen evolution favours phenotypes that increase their transmission and fitness within both vectors and hosts (Powell, 2019). Increases in vector biting rates, for example, would benefit vector-borne pathogens by boosting the number of hosts to which the pathogen gets transmitted. Increases in vector feeding persistence (i.e. continued feeding attempts when prevented from feeding or disturbed by the host) should also promote transmission to multiple hosts by enhancing vector biting rates (Rogers and Bates, 2007). At the same time, these behaviours may benefit the vector as they may lead to enhanced resource acquisition from blood meals. Nonetheless, there are costs associated with increasing biting rates for vectors since this strategy should raise the probability of vector death from host defence behaviours. Therefore, strategies that reduce chances of vector death early during the infection by preventing blood meals and increase vector feeding behaviour after parasites reach the infective stages are advantageous for pathogens. Indeed, Cator *et al*. (2013) observed this pattern when investigating temporal changes in attraction towards hosts in mosquitoes following infection by *Plasmodium*.

Further, malaria parasites should also benefit from modulating densities of gametocytes (i.e. parasite sexual stage that precedes vector development) circulating in host blood (Churcher *et al*., 2015). Such adjustments to gametocyte densities and parasitaemia can be shaped by the biting behaviour of vectors in malaria parasites. For instance, the parasite cycle frequently matches the peak of activity of their vectors (e.g. malaria and microfilaria parasites), which favours parasite transmission (Hawking, 1967; Hawking *et al*., 1968). Cornet *et al*. (2013) have demonstrated that avian malaria parasites infecting birds exposed to mosquito bites achieve higher parasitaemia than non-exposed ones. Likewise, hosts previously subjected to vector bites are more likely to successfully infect new vectors (Isaïa *et al*., 2020), suggesting *Plasmodium* may increase gametocyte production in response to mosquito bites – enhancing their own transmission. Similarly, *Leishmania*-infected sand flies display increased feeding persistence when harbouring peak levels of the parasite's infective stage (Rogers and Bates, 2007). Infected sand flies usually take an incomplete blood meal, meaning they are likely to engage in further host seeking and feeding. These studies demonstrate how pathogens may evolve to manipulate vectors or change their own development schedule within hosts to increase their success of transmission and complete their life cycle.

According to the ‘parasite manipulation hypothesis’, pathogens often evolve to manipulate their hosts' and vectors' behaviour for increased transmission and performance (Moore, 2002; Poulin *et al*., 2005). It is advantageous for pathogens that uninfected vectors are particularly attracted to infected hosts, while infected vectors ‘should’ be more attracted to uninfected hosts, as these attraction patterns would lead to higher transmission rates. Pathogens can modify host attractiveness to vectors; however, there is evidence both in support and against the manipulation hypothesis (Santiago-Alarcon and Ferreira, 2020; Yan *et al*., 2021). Previous studies on human malaria have supported this hypothesis, showing that *Anopheles* mosquitos are more attracted to infected people (Yan *et al*., 2021). Likewise, Chelbi *et al*. (2021) observed that *Leishmania*-infected hosts are more attractive to sand flies. This phenomenon could be potentially explained by the increased emission of olfactory attractants from infected hosts (Yan *et al*., 2021). Nonetheless, for birds, contradictory results have been reported with existing research suggesting either an increase, decrease or no effect of host infection status on *Culex* mosquitos feeding or attraction to hosts (Santiago-Alarcon and Ferreira, 2020). However, avian and mammalian malaria are transmitted by distinct mosquito genera, which may also explain the difference in host attractiveness as a function of infection status.

Another important trait determining pathogen performance is pathogen load, which may follow an optimal developmental schedule in the host and within the vector (Frank, 1996; Elliot *et al*., 2003; Powell, 2019). Vector-borne pathogens rely on their vectors for transmission and dispersal instead of only relying on their host as do most horizontally transmitted pathogens. Therefore, these pathogens should evolve to have low virulence, or even avirulence, to their vectors because of their critical role in transmission (Elliot *et al*., 2003). At the same time, higher parasitaemia in the vertebrate hosts, which usually correlates with higher virulence, can be selected for as higher rates of pathogen replication generally enhance transmission to vectors (Ferguson *et al*., 2003*a*; Powell, 2019). The use of vectors uncouples pathogen transmission success from host fitness and therefore weakens selection against high virulence. Nonetheless, selection should prevent excessive virulence as the host must be kept alive long enough to pass the infection to new uninfected vectors (Ewald, 1995; Frank, 1996). Additionally, vectors may incur reduced mobility or even succumb to infection if pathogen loads in the vertebrate host are too high (Ferguson *et al*., 2003*a*; Gutiérrez-López *et al*., 2019). Vector-borne pathogens face a trade-off between maintaining high parasitaemia and the survival and performance of their host and vector. However, since parasitaemia is not the only predictor of virulence and vector performance, changes in other pathogen traits might also be selected (e.g. production of toxic metabolites). Pathogens face multiple evolutionary trade-offs; the maximization of their development and replication must be balanced against multiple behavioural (e.g. vector preference towards certain hosts), immune (e.g. haemolysis of infected and uninfected erythrocytes) and physiological (e.g. blood type) traits of their hosts and vectors.

## Integrating selection across the host–vector–pathogen triad

Vector-borne transmission comes with multiple trade-offs for pathogens. While some of them can enhance their transmission and/or dispersal and increase their replication rates, they must overcome the challenge of infecting 2 distinct types of hosts. For this reason, any external factor impacting vector or host biology might disrupt pathogen development (e.g. insecticide use, see Box 1). Since vectors are ectothermic organisms that rely on precipitation and moderate/high temperatures for their own development (Forattini, 1995), pathogen development and transmission can be directly constrained by local climatic conditions (Lapointe *et al*., 2010). Vector-borne diseases such as human malaria and yellow-fever are more common in the tropics or subtropics, and, unlike other pathogens that require a single endothermic species for their transmission (e.g. SARS-CoV-2), the geographical expansion of vector-borne pathogens requires the presence of suitable vertebrate hosts, vectors and adequate climatic conditions. Naturally, populations of the same host species inhabiting different regions of the globe evolve under distinct disease pressures. One of the best-known examples of this phenomenon is the variation in the frequency of malaria resistance alleles among human populations; genes that confer protection can attain 100% prevalence among host populations in endemic areas and be absent from populations in temperate regions (Hedrick, 2011). Thus, vector-borne transmission has probably exerted distinct evolutionary pressures among distinct human (and potentially many other wildlife species) populations across the globe by constraining parasite expansion.

Box 1. Effects of human activities on pathogen and vector evolution

**Fig. 1.**
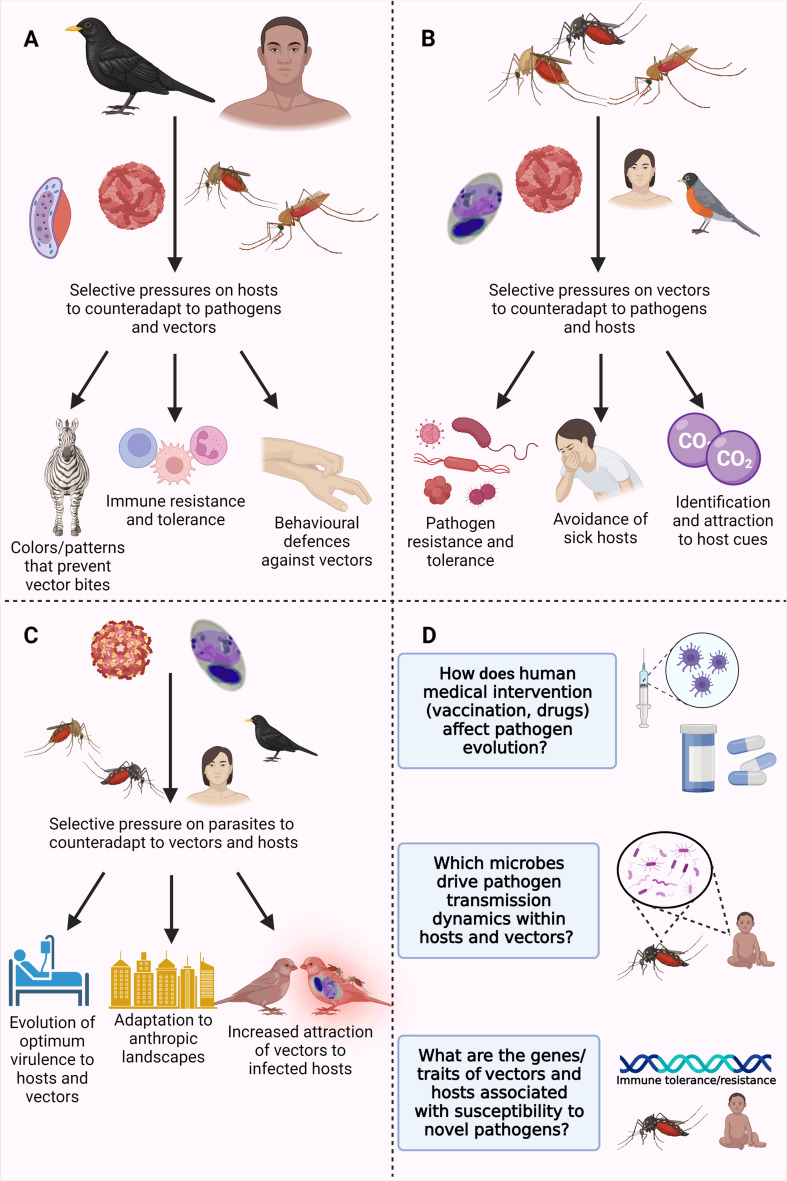
Illustration of the main selective pressures acting on hosts (A), vectors (B) and parasites (C), and examples of research questions that still lack answers (D). Figure created with BioRender.com.

**Fig. 2.**
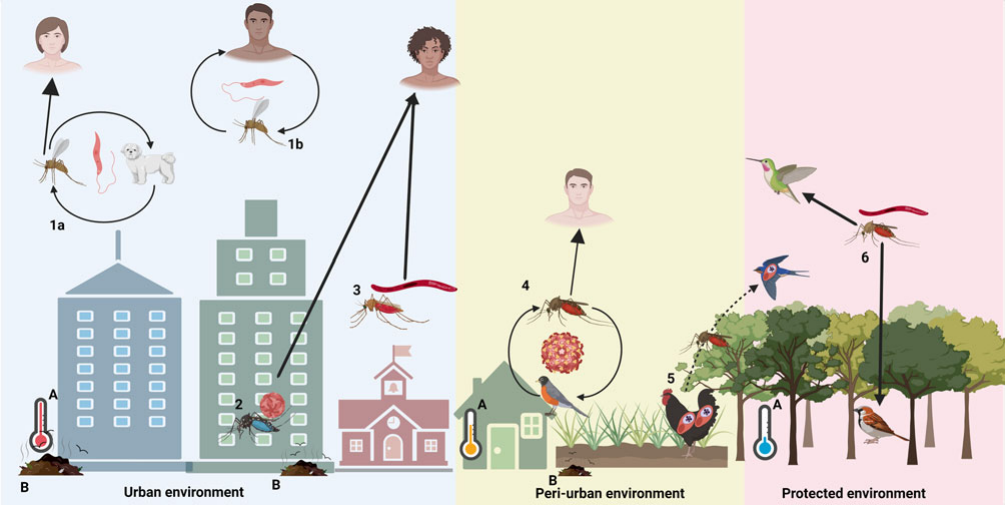
Vector-borne pathogens and their vertebrate hosts and dipteran vectors transmitted in human-modified habitat. (1) *Leishmania* spp. infects humans *via* sandfly bites in (a) zoonotic cycles (using domestic dogs as the main reservoirs) and in (b) anthroponotic cycles (i.e. human-to-human transmission). (2) Dengue virus infects mostly humans and is vectored by the mosquito *Aedes aegypti*. (3) Human malaria parasites are transmitted among humans by *Anopheles* mosquitoes in residential and agricultural areas. (4) West Nile virus circulates among birds and is vectored by *Culex* mosquitoes and infects humans mainly in residential and in agricultural areas. (5) Spillover of pathogen from domestic to wildlife animals, here illustrated by the spillover of *Plasmodium juxtanucleare* from domestic chickens to wild birds (Ferreira-Junior *et al*., 2018). (6) Avian haemosporidian prevalence has been positively and/or negatively associated with anthropization depending on the parasite genera (e.g. *Plasmodium* or *Haemoproteus*), the type of anthropic impact (e.g. farming, urbanization, pollution, etc.) and the geographic region of the study (e.g. Neotropics, Europe, etc.). Urbanization and landscape modifications driven by human activities can have several environmental effects, such as increases in (A) temperature and (B) environmental pollution. Figure created with BioRender.com.

Habitat modification (e.g. increases in temperature and environmental pollution) and scientific advances (e.g. vaccines and the development of antiparasitic medications) can directly alter vector-borne pathogen evolution by changing the taxonomic composition and abundance of mosquito communities (Forattini, 1995; Ferreira *et al*., 2016), or by altering pathogen circulation within wildlife populations, respectively (Bonneaud *et al*., 2009; Loiseau *et al*., 2010; Fecchio *et al*., 2021) (Fig. 2). Examples of human interventions that can impact pathogen and vector evolution are:

1. *Composition of hosts and vectors in urbanized areas*. Urban environments support high densities of hosts and adapted vector species that will inevitably promote pathogen specialization towards human, domestic and synanthropic wild animals under periurban conditions (Harhay *et al*., 2011; Kilpatrick, 2011; Santiago-Alarcon, 2022), and specific urban-adapted vectors (only 0.1% of all vector species occur in urban habitats (Powell, 2019; Figs 1C and 2).
2. *Unique human habits*. Certain human habits, such as housing and the use of mosquito nets and insecticides, can decrease opportunities for vectors to reach and infect hosts, further exacerbating the selective pressures acting on pathogens and vectors in human-modified environments (Fig. 2).
3. *Development of drugs and vaccines*. Access to medicine and vaccines creates an extra selective pressure for pathogens, which must overcome the effects of drugs and/or vaccine immunization to complete their life cycle. Those interventions tend to select pathogen strains that are resistant to the drugs or that make their hosts infectious before the onset of symptoms.

Hosts, vectors and pathogens impose distinct and, frequently, contrasting selective pressures on each other. For instance, vectors and parasites benefit from vectors' host-seeking behaviour and blood meal ingestion, whereas hosts may suffer from being exposed to these parasites. As a result, hosts have evolved mechanisms to avoid vectors, such as defensive and antisocial behaviours and colours and odours repellent to vectors, to escape the disease agents they carry (Fig. 1A). Vectors have evolved multiple sensory organs to detect and select their hosts based on the cues they emit (e.g. carbon dioxide detection, chemical receptors and visual stimuli) (Lucas-Barbosa *et al*., 2021) (Fig. 1B). Parasites may manipulate their hosts and vectors to increase both attraction of uninfected vectors towards infected hosts and the number of blood meals taken by an infected vector, thereby improving their own transmission (Fig. 1C). High virulence in vertebrate hosts also increases the susceptibility of infected hosts to vector feeding by minimizing defensive behaviours (Ewald, 1995). It is important to note that reductions in prevalence or parasite load among hosts and vectors can be advantageous for both to avoid the deleterious effects of infection. For example, both hosts and vectors benefit from the vectors' ability to distinguish and feed preferentially on uninfected hosts, as this can ultimately decrease the probability of infection among hosts. Thus, parasite manipulation must overcome both vector and host counter-adaptations (e.g. host resistance).

Currently, disease spread is a major threat to naïve wildlife (Daszak *et al*., 2000; Atkinson *et al*., 2014; Liao *et al*., 2017). For example, avian malaria and malaria-like parasites have excelled as one of the biggest threats to several bird species worldwide (Banda *et al*., 2013; Vanstreels *et al*., 2016; Ricklefs, 2017; McClure *et al*., 2020). This often happens due to the lack of coevolution, and thus coadaptation, between hosts and pathogens. However, pathogen tolerance can emerge in some populations of highly susceptible naïve species (Atkinson *et al*., 2013). Furthermore, past research has showed that facilitated adaptation (i.e. mediated by human intervention) can shape the odds of susceptible species/population extinctions in nature (Samuel *et al*., 2020). Hosts genetically modified to be resistant to infections could reduce a species' probability of extinction over time and place those hosts 1 step ahead in the evolutionary arms race against their pathogens (Samuel *et al*., 2020). Therefore, natural or facilitated adaptation leading to resistant or tolerant phenotypes might represent the main tool for susceptible hosts to persist when facing the introduction of new deleterious pathogens.

Immune response is an important mechanism deployed by both hosts and vectors to resist or tolerate infections (Hoffmann *et al*., 1999; Mendonça *et al*., 2013; Maitre *et al*., 2022). Vectors and hosts have evolved multiple immune pathways against infection, and both hosts and vectors would benefit indirectly from each other's defences against parasites if these defences were efficient enough to reduce pathogen circulation within a region. At the same time, vector pathogen inhibition tends to increase selective pressures for pathogens that can overcome the vector's immune system. Because vector-borne pathogens often induce lower virulence in their vectors (Elliot *et al*., 2003), the selective pressure to mount strong immune responses is certainly more evident among vertebrate hosts. For instance, humans use a robust combination of innate and adaptive immune responses against malaria parasites (Mourão *et al*., 2020). Nonetheless, the host's immune system can act against vectors as well, as some host immunoglobulins can remain active for days, targeting parasites within the vector's midgut and having deleterious effects on, or even causing the death of vectors (Maitre *et al*., 2022). For this reason, vaccines against vector-borne diseases could target vector survival to reduce pathogen transmission. Host immunoglobulins produced in response to immunization against commensal bacteria inhabiting the vector's midgut can alter the vector's gut microbiome, which can potentially reduce vector fitness and/or competence (Aželytė *et al*., 2022). Optimizing novel immunization strategies against vectors, pathogens and their microbiomes could exert new evolutionary pressures on vector-borne pathogens.

Vector and host microbiomes can also have profound effects on other facets of vector, host and pathogen interactions. This happens because the cascading effects of gut microbiome disruption can alter not just vector development but also the parasite cycle through indirect effects on co-occurring microbes and, indirectly, pathogen transmission rates (Dennison *et al*., 2014). For instance, vector microbiomes can alter vector competence due to resource competition or by mediating vector immune responses (Dennison *et al*., 2014). In addition, skin microbes are known to influence hosts' attractiveness to vectors (Fredrich *et al*., 2013; Verhulst *et al*., 2018) and a higher diversity of skin microbes seems associated with limited vector attractiveness, thereby providing protection against vector-borne diseases (Lucas-Barbosa *et al*., 2021). At the same time, the over exposure to antibiotics (due to direct medical intervention or indirect exposure to antibiotics used in farms and croplands) decreases gut and skin microbiome diversity and alters immune responses (Francino, 2016; Raymann *et al*., 2018). Hence, excessive use or exposure to antibiotics could increase human and wildlife attractiveness to vectors and their susceptibility to pathogens.

Different pathogens may compete or have synergistic interactions among them within their hosts and/or vectors (Clark *et al*., 2016, 2020). In the first case, competition might decrease fitness of 1 or more pathogens by limiting host resources available (Harvey *et al*., 2011; Clark *et al*., 2016). At the same time, prior infections might facilitate the development of new pathogens due to the weakening of the host immune system generated by the primary infection, favouring pathogens in secondary infections (Vaughan and Turell, 1996; Pollitt *et al*., 2015). Indeed, Clark *et al*. (2016) have observed altered heterophil/lymphocyte rates among birds coinfected by microfilaria and haemosporidians, indicating this nematode could facilitate protozoan infections as a result of immune modulation. Overall, there is increasing evidence that vector–host–pathogen interactions are mediated by several other players associated directly or indirectly with the pathogen's cycle (e.g. symbiotic microbes and other pathogens) (Vaughan and Turell, 1996; Dennison *et al*., 2014; Jupatanakul *et al*., 2014; Pollitt *et al*., 2015; Verhulst *et al*., 2018).

Environmental changes associated with human activities also represent a selective force-driving pathogen and vector evolution (see Box 1). For vector-borne pathogens, temperature, precipitation and distance to water bodies are major drivers of pathogen prevalence due to their direct effects on vector development (Ferraguti *et al*., 2018, 2020). Anthropogenic landscapes present a distinct microclimate, partly because they often attain higher temperatures, which can affect both the abundance and richness of vectors (Ferraguti *et al*., 2016) and, hence, favour the transmission of pathogens able to develop in the few vector species remaining. In addition, other human landscape interventions can shape vector evolution, such as larvicide treatments, which have been implemented in many areas to regulate mosquito populations. These interventions select for resistant/tolerant strains of mosquitoes and exert pressures on pathogens due to constraints on the numbers of available vectors (Ferraguti *et al*., 2020). However, ultimately, our unique human talent to create synthetic drugs and vaccines is probably the most promising weapon against pathogens and their vectors.

Scientists have developed drugs and vaccines that have greatly reduced the prevalence and even eradicated certain diseases, such as smallpox. However, vaccines have been successfully developed only for very few vector-borne diseases, such as yellow fever, dengue and Japanese encephalitis (Olajiga *et al*., 2021). There are current initiatives to develop and/or improve vaccines for other important human vector-borne diseases, such as leishmania and malaria (Lage *et al*., 2020; Datoo *et al*., 2021), but major advances have been few and far between. Remarkably, World Health Organization recommended in October 2021 the use of an RTS,S/AS01 malaria vaccine among children inhabiting regions of moderate-to-high transmission risk *of P. falciparum* malaria infection. Nonetheless, this vaccine confers only modest protection against malaria infections (Laurens, 2020). Furthermore, use of vaccines can promote increase of parasite virulence in naïve hosts over time due to relaxed selective evolutionary pressures on host mortality (Gandon *et al*., 2001). The development of new vaccines and drugs could become a strong tool to control or even eradicate vector-borne diseases. Because scientific advances may occur faster than biological evolution, they represent the best option to overcome pathogens and allow hosts to surge ahead in the coevolutionary arms race (Powell, 2019).

## Conclusion

Here, we summarized the main evolutionary pressures faced by hosts, vectors and pathogens associated with vector-borne transmission (see Fig. 1A–C and Table 2). Pathogens and their hosts evolve in tandem and, consequently, adaptation by 1 antagonist should result in a counter-adaptation by its counterpart. In the specific case of vector-borne pathogens, 3 distinct ‘players’ coevolve together and are impacted by direct or indirect selective pressures from the others. Generally, vertebrate hosts and vectors should evolve traits allowing them to experience only reduced infection rates and infection-mediated fitness losses *via* increased resistance and/or tolerance to infections. Nevertheless, strategies towards less pathogenic interactions are highly variable between those 2 groups. While hosts are passively infected by parasites and should, therefore, evolve towards less attractive phenotypes, vectors would benefit from an active avoidance of infected hosts. Despite the fact pathogens are more virulent to their hosts than to their vectors, both have evolved immune/biochemical mechanisms to combat infections. On the other hand, parasites have evolved multiple mechanisms to increase their own transmission (e.g. behavioural manipulation, high rates of replication, etc.) and, due to their undoubtedly faster evolutionary rates compared to both hosts and vectors, parasites are unlikely to be overtaken naturally by either their vertebrate or vector hosts in this tripartite coevolutionary arms race. Thus, scientists should consider the evolutionary context encompassing hosts, vectors, pathogens and their microbiome to create new effective pathways for treatments and preventive interventions (see Fig. 1D), which could minimize pathogen burden for wildlife and human populations.

**Table 2.** Examples of studies on adaptations and counter-adaptations of hosts, vectors and pathogens

Host sp.	Vector sp.	Pathogen ID	Topic	Main results	Reference
Zebra (*Equus quagga*)	Horseflies (*Haematopota pluvialis* and *Tabanus bromius*)	None	Factors that protect hosts from vector bites	Stripes prevent landing and host approach by flies (i.e. protective effect)	How *et al*. (2020)
Humans (*Homo sapiens*)	None	*Plasmodium falciparum*	Host immune resistance and tolerance to pathogens	Blood group ‘O’ represents a protective phenotype against severe infections whereas ‘A’ blood group is associated with higher pathogenicity	Goel *et al*. (2015)
Rock pigeons (*Columba livia*)	Flies (*Pseudolynchia canariensis*)	*Haemoproteus columbae*	Host behavioural defences against vectors	Anti-vector behaviour and immune reaction decrease fly fitness and survival but do not affect pathogen prevalence	Waite *et al*. (2014)
None	Mosquitoes (*Anopheles* spp.)	*Plasmodium* spp.	Vector immune resistance and tolerance to pathogens	Vectors can constrain parasite development by degrading sporozoites when these migrate to the salivary glands through the haemolymph	Hillyer *et al*. (2007)
Great tits (*Parus major*)	Mosquitoes (*Culex pipiens*)	*Plasmodium* spp.	Vector avoidance of infected hosts	Vectors avoided infected hosts^a^	Lalubin *et al*. (2012)
Humans (*H. sapiens*)	Sandflies (*Lutzomyia* spp.)	None	Identification and attraction to host cues	Vectors were attracted to carbon dioxide and human odour	Pinto *et al*. (2001)
Mice	Mosquitoes (*Anopheles stephensi*)	*Plasmodium chabaudi*	Optimum virulence towards hosts and vectors	Pathogen benefits from high virulence in hosts but lower virulence among vectors	Ferguson *et al*. (2003*a*)
Dogs (*Canis familiaris*)	Sandflies (*Phlebotomus perniciosus*)	*Leishmania infantum*	Pathogen manipulation of hosts/vectors	Pathogen induces physiological modifications in the host that increase their attractiveness to their vectors	Chelbi *et al*. (2021)
Mice	Sandflies (*Lutzomyia longipalpis*)	*Leishmania* spp.	Pathogen manipulation of hosts/vectors	Pathogen induces increase in feeding persistence among infected vectors	Rogers and Bates (2007)

a Many studies on this topic show no or contrary effects.
